# Gene expression profile data of the developing small intestine of *Id2*-deficient mice

**DOI:** 10.1016/j.dib.2019.103717

**Published:** 2019-03-15

**Authors:** Kentaro Mori, Kota Tamada, Hisanori Kurooka, Makoto Matsui, Toru Takumi, Yoshifumi Yokota

**Affiliations:** a Department of Neurology, Kanazawa Medical University, 1-1 Uchinada, Ishikawa 920-0293, Japan; b Division of Molecular Genetics, Department of Biochemistry and Bioinformative Sciences, School of Medicine, Faculty of Medical Sciences, University of Fukui, 23-3 Matsuoka-Shimoaizuki, Eiheiji, Fukui 910-1193, Japan; c RIKEN Brain Science Institute, 2-1 Hirosawa, Wako, Saitama, 351-0198, Japan; d Faculty of Nutritional Science, Department of Nutritional Management, Sagami Women's University, Sagamihara, Kanagawa, 252-0383, Japan

**Keywords:** Endoderm, Foregut, Midgut, *Id2*, *Sox21*, Microarray

## Abstract

This article contains data related to the research article entitled “Id2 determines intestinal identity through repression of the foregut transcription factor, Irx5” [1]. *Id2* deficient (*Id2*^−/−^) mice developed gastric tumors and heterotopic squamous epithelium in the small intestine. These tumors and heterotopic tissues were derived from ectopic gastric cells and squamous cells formed in the small intestine respectively during development. In this study, microarray data of the developing small intestine of *Id2*^−/−^ mice was analyzed.

**Table:** Specifications table

Subject area	*Developmental Biology, Gastroenterology*
More specific subject area	*Gene expression*
Type of data	*Table, Figure*
How data was acquired	*Applied Biosystems Mouse Genome Survey Microarray Ver2.0*
Data format	*Raw and Normalized*
Experimental factors	*The midgut of E13.5 Id2KO and wild-type embryos*
Experimental features	*Microarray expression profile analysis of Id2KO midgut*
Data source location	*University of Fukui, 23-3 Matsuoka-Shimoaizuki, Eiheiji, Fukui, Japan*
Data accessibility	*Microarray data are available from Gene Expression Omnibus database. The deposited data can be found at:**https://www.ncbi.nlm.nih.gov/geo/query/acc.cgi?acc=GSE43014*
Related research article	*Mori K, Nakamura H, Kurooka H, Miyachi H, Tamada K, Sugai M, Takumi T, Yokota Y. 2018. Id2 determines intestinal identity through repression of the foregut transcription factor Irx5. Mol Cell Biol 38:e00250-17.* *https://doi.org/10.1128/MCB.00250-17**.**[1]*

**Table:** 

**Value of the data** • These data provide information about the cellular differentiation of the developing gastrointestinal tract. • These data give insight into Id2 regulated foregut gene expression in the midgut. • These data are useful for understand the molecular mechanisms underlying gastrointestinal organ development. • The midgut of Id2 knockout mice is useful for identifying master regulator of gastric epithelial cell differentiation which has not yet been identified. These data can also be a benchmark to elucidate the function of such factors.

## 1 Data

Microarray analysis was performed in the developing small intestine of *Id2*^−/−^ mice. In total, 34 genes were differentially expressed in *Id2*^−/−^ embryo compared with *Id2*^+/+^ embryo with criteria of fold change >2. Of these differentially expressed genes, 14 genes were upregulated and 20 genes were downregulated in *Id2*^−/−^ embryo (Table 1) [1].

**Table 1:** Differentially expressed genes in *Id2* KO midgut.

	Gene	Expression pattern in the developing digestive tract	Reference
Up-regulated genes (KO/WT, fold change >2)	*Cym*, *Irx3*, *Irx5*	Specifically expressed in foregut endoderm	[2], [3]
	*Krt15*, *Foxa2*, *Adcy8*	Preferentially expressed in foregut endoderm	[4], [5], [6]
	*Traf6*	Oral endoderm and mesenchyme	[7]
	*Orfr1337*, *Cacng7*, *Wdr86*, *Ocrl*, *C030016D13Rik*, *Cdc96*	not anotated	
Down-regulated genes (KO/WT, fold change <0.5)	*Sul1d1*, *Spink3*, *Anxa13*, *Muc13*, *Lingo1*, *Bspry*, *Fabpl*	Highly expressed in midgut endoderm	[8], [9], [10], [11], [12], [13], [14]
	*Cbln2*	Preferentially expressed in midgut mesenchyme	[13]
	*Myl1*, *Slc27a2*, *Foxq1*	Highly expressed in the other region of midgut endoderm	[13], [15]
	*Them7*, *Kynu*, *Ppp1r1b*, *Mkrn2os*, *Hapln2*, *BC030870*, *2610044O15Rik*, *Ifi203*, *Faim3*	not anotated	

**Fig. 1:**
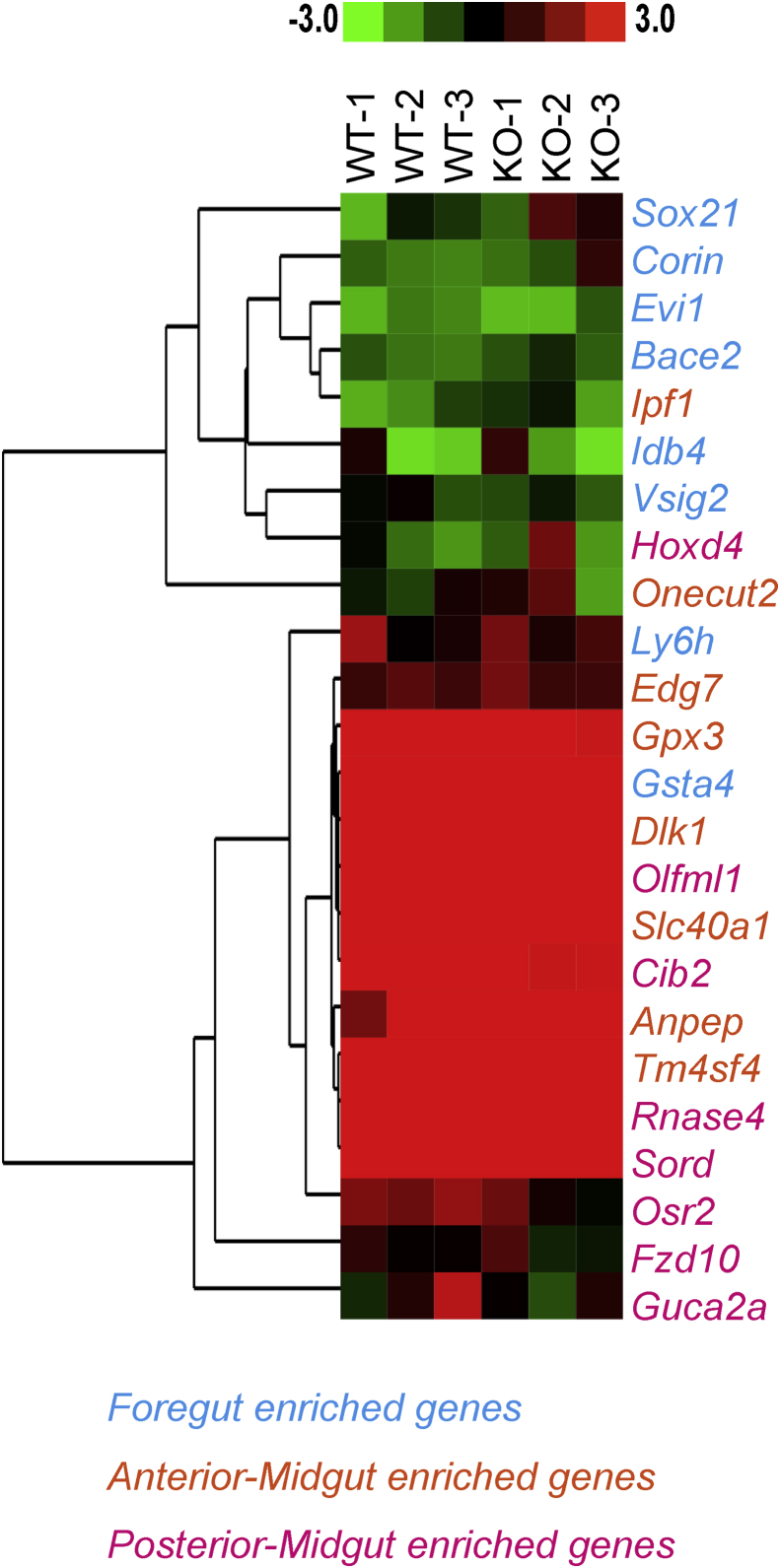
Heatmap of specific gene expressions in the midgut of *Id2* wild-type (WT) and *Id2* deficient (KO) mice embryos. The colored scale at the top of heatmap is log based. Genes are preferentially expressed in the specific gut segment. Foregut enriched genes, Anterior-Midgut enriched genes and Posterior-Midgut enriched genes were represented with different colors; cyan, orange and magenta respectively. Hierarchical clustering was performed with the complete-linkage method.

Furthermore, the expression levels of the selected 24 genes that are preferentially expressed in a specific embryonic gut segment, including foregut (eight genes), anterior-midgut (eight genes) and posterior-midgut (eight genes) were analyzed [16]. Heatmap visualization indicated that the expression of six of foregut-enriched genes were upregulated in *Id2*^−/−^ embryo (Fig. 1) while the expressions of three of the midgut-enriched genes were remarkably downregulated in *Id2*^−/−^ embryo. The remaining two foregut-enriched genes and 12 midgut enriched genes were not altered.

**Fig. 2:**
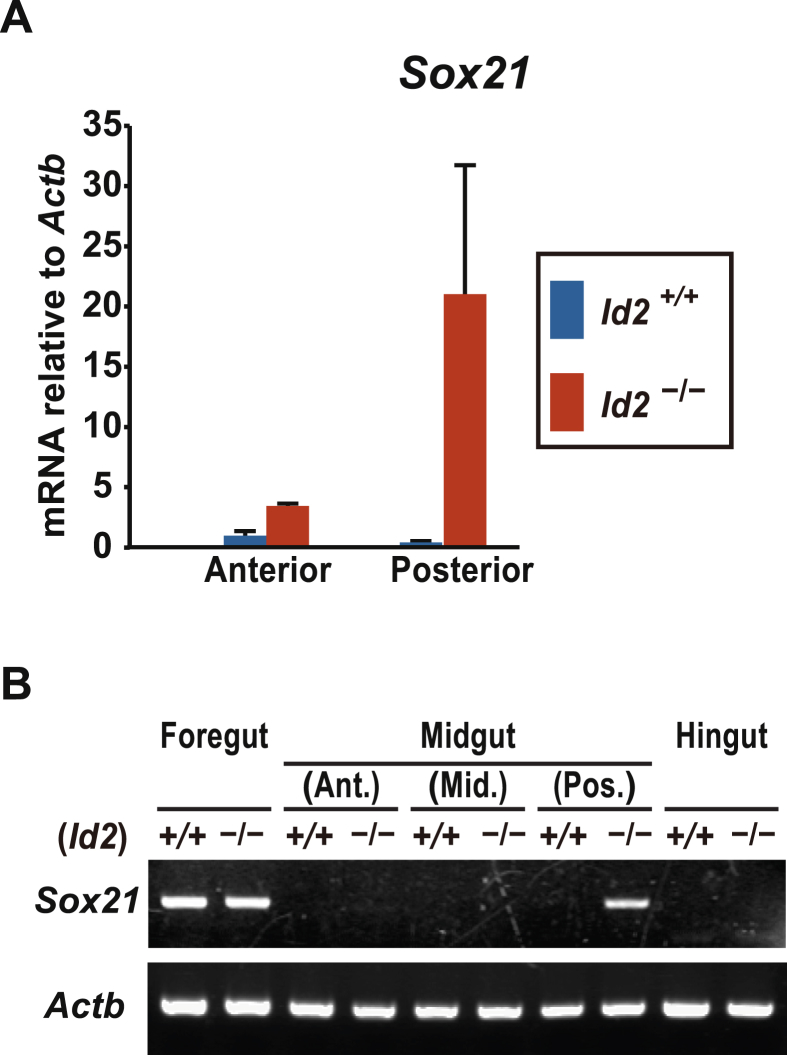
*Sox21* expression in the developing gastrointestinal tract of *Id2*^−/−^ embryo. (**A**) qRT-PCR analysis of *Sox21* expression in E13.5 midguts. Midgut tissues were subdivided into anterior and posterior parts (n=7 per genotype). (**B**) RT-PCR analysis of *Sox21* expression in E15.5 gastrointestinal tract. Midgut tissues were subdivided into three segments along the anterior-posterior axis. Ant - anterior segment of midgut; Mid - middle segment of midgut; Pos - posterior segment of midgut.

*Sox21* is highly expressed in the anterior region over the period of foregut endoderm formation [3], [16], [17]. qRT-PCR analysis revealed that *Sox21* expression increased only in the posterior part of the *Id2*^−/−^ mice midgut (Fig. 2A). RT-PCR analysis clearly showed that heterotopic *Sox21* expression was confined to the midgut of *Id2*^−/−^ embryo, but not to the posterior part of midgut or hindgut (Fig. 2B).

## 2 Experimental design, materials and methods

### 2.1 Animals

*Id2* mutant mice with 129/Sv genetic background were used for analysis [18]. Preparation of *Id2*^+/+^ and *Id2*^−/−^ embryos was performed by crossing 8-week-old *Id2*^+/−^ male and *Id2*^+/−^ female mice.

### 2.2 RNA extraction

Total RNA samples were extracted using an RNeasyMini Kit (QIAGEN, Valencia, CA, USA). Tissue lysate was purified by QIAshredder (QIAGEN) and treated with DNaseI to remove genomic DNA. For microarray analysis, total RNA from three midguts of the same genotype was taken as one sample. RNA quality was measured using the Agilent 2100 Bioanalyzer 2100 (Agilent Technology, Wilmington, DE, USA), and samples with 28S/18S ribosome ratio >2.0 were used for analysis.

### 2.3 Microarray

One microgram of total RNA was amplified and labeled with digoxigenin (DIG) for one round using a NanoAmp RT-IVT Labeling Kit (Applied Biosystems, Foster City, CA, USA). DIG labeled cRNA was fragmented and hybridized to Mouse Genome Survey Microarray ver.2.0 (Applied Biosystems) followed by chemiluminescence detection.

### 2.4 Data analysis

Raw signal values were normalized by the median. In all probe sets with false spots (flag < 5000) and signal-to-noise values < 3 (as determined by the software) were excluded. Normalized signal values were converted to log_2_ ratios. Fold changes between *Id2*^−/−^ and wild-type samples were calculated for each of the resulting probe sets. Heatmap visualization was constructed by Cluster 3.0 and Treeview software [19].

### 2.5 RT-PCR

Oligo(dT)-primed cDNA synthesis was performed using SuperScript III reverse transcriptase (Invitrogen, Carlsbad, CA, USA). qRT-PCR was performed using the Power SYBR green PCR master mix and a StepOnePlus real-time PCR system (Applied Biosystems, Foster City, CA, USA). Primer sequences for RT-PCR analysis are as follows: *Sox21*-forward, TACATGATCCCGTGCAACTG; and *Sox21*-reverse, TTCGAGCTGGTCATTCACTG. PCR primer sequences for qRT-PCR and *Actb* primers for RT-PCR analysis were described previously [1].

## Funding

This work was supported in part by JSPS KAKENHI (JP24790194 to K.Mori, JP 21390092 and JP24390077 to Y.Yokota, JP16H06316 and JP16H06463 and JP16K13110 to T.Takumi) and by JST CREST (to T.Takumi) and by the grant provided by The Ichiro Kanehara Foundation (to K. Mori) and by research grants from the University of Fukui (to Y.Yokota).
